# Association of Plasma Lipids and Polar Metabolites with Low Bone Mineral Density in Singaporean-Chinese Menopausal Women: A Pilot Study

**DOI:** 10.3390/ijerph15051045

**Published:** 2018-05-22

**Authors:** Diana Cabrera, Marlena Kruger, Frances M. Wolber, Nicole C. Roy, John J. Totman, Christiani Jeyakumar Henry, David Cameron-Smith, Karl Fraser

**Affiliations:** 1 School of Food and Nutrition, Massey University, Tennent Drive, Palmerston North 4442, New Zealand; M.C.Kruger@massey.ac.nz; 2 Food Nutrition & Health Team, Food & Bio-Based Products Group, AgResearch Grasslands, Palmerston North 4442, New Zealand; nicole.roy@agresearch.co.nz (N.C.R.); d.cameron-smith@auckland.ac.nz (D.C.-S.); karl.fraser@agresearch.co.nz (K.F.); 3 Riddet Institute, Massey University, Palmerston North 4442, New Zealand; 4 Centre for Metabolic Health Research, Massey University, Tennent Drive, Palmerston North 4442, New Zealand; F.M.Wolber@massey.ac.nz; 5 High-Value Nutrition National Science Challenge, Auckland 1142, New Zealand; 6 A*Star-NUS Clinical Imaging Research Centre, Singapore 117599, Singapore; John_Totman@circ.a-star.edu.sg; 7 A*Star-NUS Clinical Nutrition Research Centre, Singapore 117599, Singapore; jeya_henry@sics.a-star.edu.sg; 8 The Liggins Institute, The University of Auckland, Auckland 1142, New Zealand

**Keywords:** metabolomics, lipidomics, osteoporosis, menopause, biomarkers

## Abstract

The diagnosis of osteoporosis is mainly based on clinical examination and bone mineral density assessments. The present pilot study compares the plasma lipid and polar metabolite profiles in blood plasma of 95 Singaporean-Chinese (SC) menopausal women with normal and low bone mineral density (BMD) using an untargeted metabolomic approach. The primary finding of this study was the association between lipids and femoral neck BMD in SC menopausal women. Twelve lipids were identified to be associated with low BMD by the orthogonal partial least squares (OPLS) model. Plasma concentrations of eight glycerophospholipid, glycerolipid, and sphingolipid species were significantly lower in menopausal women with low BMD but higher in two glycerophospholipid species (phosphatidylinositol and phosphatidic acid). Further, this study found no significant differences in plasma amino acid metabolites. However, trends for lower 4-aminobutyric acid, turanose, proline, aminopropionitrile, threonine, and methionine were found in women with low BMD. This pilot study identified associations between lipid metabolism and femoral neck BMD in SC women. Further studies are required on larger populations for evaluating the bone health effect of these compounds and their usefulness as clinical biomarkers for osteoporosis prediction in women.

## 1. Introduction

Menopausal women have a greater risk of bone loss and developing osteoporosis. Osteoporosis affects above 200 million people worldwide, and about 9 million osteoporotic fractures, of which 1.6 million are at the hip, are registered per year [1]. In Asian populations, hip fractures account for around 30% of those worldwide, and in Singapore, hip fracture rates have increased by 1.2% annually in Chinese women [2].

In women, low oestrogen level is a risk factor for osteoporosis. Oestrogen withdrawal promotes the activation of bone remodelling at the basic multicellular units (BMUs). Bone formation decreases because there is reduction of osteoblastic cell lifespan, and bone resorption increases as a result of increased differentiation and lifespan of osteoclasts [3]. The impact of oestrogen loss in bone metabolism, including several biochemical and physiological alterations, is characterized by high levels of oxidative stress, inflammation, and altered metabolism in the bone microenvironment [4,5]. Changes in lipid and polar metabolites have been associated with oxidative stress and inflammation affecting bone metabolism. Previous studies demonstrated that oxidative stress, caused by mitochondrial alterations, induces reactive oxygen species (ROS) generation, and leads to osteoblast cell death by increasing the oxidized bone microenvironment [6,7]. In addition, oestrogen withdrawal upregulates bone microenvironment pro-inflammatory cytokines like interleukin-1 (IL-1), interleukin-6 (IL-6), tumor necrosis factor-α (TNF-α), granulocyte macrophage colony-stimulating factor, macrophage colony-stimulating factor (M-CSF), and prostaglandin-E_2_ (PGE_2_), which regulate osteoclast differentiation and function and therefore bone loss [8,9]. Osteoporosis may be present before diagnosis; despite current serum biochemical analysis and radiological examination methods for screening of osteoporosis and fracture risk in women [10], none of these methods are suitable for prediction of early bone loss in women. The elucidation of the cellular and biochemical events on bone metabolism after oestrogen withdrawal may lead to a better understanding of the molecular mechanisms involved in osteoporosis and bone cell signalling pathways in women, and subsequently identify early predictors of bone loss that can be used as prognostic markers.

Metabolomics offers the potential for analyzing the biochemical changes in the pathology of diseases. Metabolomic studies are conducted by using several analytical platforms; however, nuclear magnetic resonance (NMR) and mass spectrometry (MS) are the most widely reported techniques [11]. A small number of plasma metabolomic studies have reported that the metabolite concentration shifts under menopausal conditions in ovariectomized (OVX) animals and humans [12,13,14,15], while numerous studies on the relationship between bone loss and low oestrogen levels in both OVX animals and humans have been reported [16,17,18,19,20,21,22].

Because of the limited information reported on the association between the plasma metabolome and bone mineral density (BMD) in Singaporean-Chinese menopausal women (SC), research in this area is required to allow the identification of potential metabolites that can be used to understand the causal pathways involved in menopausal osteoporosis. We hypothesized that novel metabolomic markers will improve the prediction of SC menopausal women at increased osteoporotic risk. Therefore, this study aimed to analyze the lipid and polar metabolite profiles of blood plasma SC menopausal women using a liquid chromatography–mass spectrometry (LC–MS) untargeted metabolomic approach. Moreover, to maximize the identification of biomarkers associated with bone loss in menopausal women, we also performed statistical tests on a restricted subset of the SC women with either osteoporosis (T-score < −2.5) or normal BMD (T-score > −1). We hypothesized that lipids and polar metabolites of women with osteoporosis differed from the whole population. Further, correlation analyses were conducted between metabolites and femoral neck bone mineral density (BMD) in menopausal women.

## 2. Materials and Methods

### 2.1. Standards and Reagents

Formic acid, *d*_2_-tyrosine, sucrose, alanine, arginine, asparagine, aspartic acid, glutamic acid, glutamine, histidine, homoserine, isoleucine, leucine, lysine, phenylalanine, proline, serine, theanine, threonine, tyrosine, valine, amino acid standard mixture (physiological mixture), 16:0 *d*_31_-18:1 PE (phosphatidylethanolamine), and ammonium formate were purchased from Sigma–Aldrich Chemicals Co. (St. Louis, MO, USA). Ultrapure water was obtained from a Milli-Q^®^ system (Millipore, Bedford, MA, USA). Acetonitrile (ACN), methanol and isopropanol were optima LC–MS grade, chloroform was high-performance liquid chromatography (HPLC) grade, and all were purchased from Thermo Fisher Scientific (Auckland, New Zealand).

### 2.2. Study Population, Inclusion and Exclusion Criteria

All subjects were informed about the objective of the study and gave their informed consent for the participation in the present study. The study was approved by the Ethics Committee for Research Involving Human Subjects, Singapore (Approval No. 2014/01066). The study was done in accordance with the Declaration of Helsinki (2000) of the World Medical Association.

Ninety-seven SC menopausal women aged between 55 and 70 years were included in the study. The key inclusion criterion was women who were at least five years menopausal (based on a history of cessation of menstruation). Exclusion criteria included prior diagnosis with osteoporosis, diabetes mellitus or any condition that affects bone and liver function, and to not be taking any medication that will affect the study.

### 2.3. Blood Collection

Blood samples were taken only once between 8 and 10 a.m., after an overnight fast (10–12 h). Blood was collected in ethylenediamine tetraacetic acid (EDTA) tubes (BD Vacutainer^TM^ K3E 15%, Becton, Dickinson and Company, Plymouth, UK) and plasma samples transferred into separate 1 mL tubes for extractions for metabolomics analysis; C-terminal telopeptide of type I collagen (CTx-1), parathyroid hormone (PTH) and 25(OH) vitamin D3 were stored frozen at −80 °C and thawed on the day of the analysis.

### 2.4. Analysis of Blood Parameters

Blood samples were taken to measure plasma markers of CTx-1 as well as PTH and 25(OH) vitamin D3. CTx-1 and PTH concentrations were analyzed by electrochemiluminescence immunoassay using the Roche COBAS^®^ e411 system (Roche Diagnostics, Indianapolis, IN, USA). Serum 25(OH) vitamin D3 concentration was analyzed using isotope-dilution liquid chromatography–tandem mass spectrometry (IDLC–MS–MS) [23] by Canterbury Health Laboratories, Christchurch, New Zealand.

### 2.5. Bone Mineral Density

BMD was measured using dual X-ray absorptiometry (DXA). DXA scans of hip (femoral neck) were carried out using a Hologic QDR-Discovery A densitometer (Hologic Discovery QDR 4500A densitometer, Hologic Inc., Bedford, MA, USA). BMD was determined and women were classified into normal or low BMD according to the World Health Organization (WHO) classification. WHO provides an operational definition of osteoporosis based on T-score, where the T-score is the number of the standard deviation below of the mean peak BMD for young-adults. Women with a BMD 2.5 standard deviations below are classified as osteoporotic. In this study, participants in the entire cohort were classified into two groups according to bone status: (1) low BMD = women with a T-score < −1, and (2) normal BMD = women with a T-score > −1.0. Further, a selected subset of participants were also classified into two groups: (1) osteoporosis = women with a T-score < −2.5, and (2) normal BMD: women with T-score > −0.1 [24].

### 2.6. Metabolomic Analysis

#### 2.6.1. Sample Preparation

Lipids and polar metabolites were extracted from plasma using a biphasic solvent. Lipids were analyzed by reverse-phase liquid chromatography–mass spectrometry (RP LC–MS) with positive- and negative-mode electrospray ionization. Polar metabolites were analyzed by hydrophilic interaction liquid chromatography–mass spectrometry (HILIC LC–MS) with only positive-mode electrospray ionization. Briefly, plasma samples were thawed in fridge at 4 °C. Then, the samples were vortexed for 1 min. 100 µL of plasma was transferred to a microcentrifuge tube and 800 µL of cold chloroform:methanol (1:1 *v*/*v*) were added to the tubes. The tubes were agitated by hand for 1 min and the samples were stored for 30 min at -20 °C. 400 µL of water were added to each sample and vortexed for 30 s. The samples were centrifuged for 15 min at 13,913 g at 4 °C. 200 µL of the lower phase (organic phase) were taken for lipid analysis and transferred to a new microcentrifuge tube to evaporate to dryness under a stream of nitrogen. Two hundred and fifty µL of the upper phase (polar aqueous) were collected for polar metabolite analysis and transferred in to new microcentrifuge tube to evaporate to dryness under stream of nitrogen. The lower-phase tubes were reconstituted in 100 µL of Folch solvent mixture (choroform:methanol 2:1 *v*/*v*) containing 16:0 *d*_31_-18:1-PE internal standard at 10 µg/mL concentration. The upper-phase tubes were reconstituted in 300 µL of ACN:water (1:1 *v*/*v*) containing formic acid (0.1%). All the samples were vortexed for 1 min, centrifuged for 10 min 13,913 g at 4 °C and 100 µL were transferred to a vial containing an insert and stored at 4 °C for immediate polar metabolite analysis. Polar metabolite quality control (QC) samples were prepared with 60 µL of each sample and placed in a tube to be pooled. The pooled QC samples were transferred into multiple pooled QC vials. Lipid QC samples were prepared with 20 µL of each sample. The pooled lipid QC samples were aliquoted into multiple pooled quality control vials [25].

#### 2.6.2. LC-MS Conditions and Metabolite Identification

Untargeted metabolomic approach was carried out on the Thermo LC–MS system (Thermo Fisher Scientific, Waltham, MA, USA). This system consisted of an Accela 1250 quaternary pump, a Thermo-PAL auto-sampler fitted with a 15,000 psi injection valve (CTC Analytics AG., Zwingen, Switzerland). 

Lipid chromatographic separation was conducted by using a C18 column (100 × 2.1 mm; 1.7 μm particle size, Waters, Milford, MA, USA). The samples were separated with a gradient elution program and flow rate of 600 μL/min. The mobile phase was a mixture of 0.1% formic acid in ACN–isopropanol (50:50, *v*/*v*) (Solvent A), 0.1% formic acid in ACN-water (60:40 *v*/*v*) with 10 mM ammonium formate (Solvent B) and a mixture of 0.1% formic acid in isopropanol-ACN (90:10 *v*/*v*) with 10 mM ammonium formate (Solvent C). The gradient elution was conducted starting with 85% solvent B (0–1 min), decreased at 70–18% (2–11 min) and held at 1% (11.50–14 min), returned to 85% (22–27 min), while solvent A was held at 100% (14.1–22 min) and allowed to equilibrate for 5 min prior to the next sample injection. Column effluent was connected to a 2 μL injection loop and a Q Exactive Orbitrap mass spectrometer using positive and negative electrospray ionization. Data were collected in profile data acquisition mode over a mass range of *m*/*z* 200–2000 at a mass resolution setting of 70,000 with a maximum trap fill time of 100 ms using the Xcalibur software package ver. 2.2 SP1.48 provided by the manufacturer (Thermo Fisher Scientific, San Jose, CA, USA). Lipid positive ion mode parameters were as follows: spray voltage, 3.2 kV; capillary temperature, 275 °C; capillary voltage, −0.2 V, tube lens 120 V. Negative ion mode parameters were as follows: spray voltage, −3.2 kV; capillary temperature, 275 °C; capillary voltage, −0.1 V, tube lens −100 V. 

Polar metabolites chromatographic separation was conducted by using a ZIC-pHILIC column (100 mm × 2.1 mm, 5 μm; Merck, Darmstadt, Germany). The samples were separated with a gradient elution program and a flow rate of 250 μL/min. The mobile phase was a mixture of ACN-formic acid (99.9:0.1, *v*/*v*) (solvent A) and water–ammonium formate (16 mM, pH 6.3) (solvent B). The gradient elution programme was: held at 97% A (0–1 min), 97–70% A (1–12 min), 70–10% A (12–14.5 min), held at 10% A (14.5–17 min), returned to 97% A (17–18.5 min) and allowed to equilibrate for a further 5.5 min prior to the next injection. Column effluent was connected to a 20 μL injection loop and an Exactive Orbitrap mass spectrometer using positive and negative electrospray ionization. Data were collected in profile data acquisition mode over a mass range of *m*/*z* 55–1100 at a mass resolution setting of 25,000 with a maximum trap fill time of 100 ms using the Xcalibur software package provided by the manufacturer. Samples were run in positive ionization mode. HILIC positive ion mode parameters were as follows: spray voltage, 2.7 kV; capillary temperature, 275 °C; capillary voltage, 29.1 V, tube lens 108.8 V.

#### 2.6.3. Metabolite Identification and Processing

Raw LC–MS data were acquired using Xcalibur v2.1 software (Thermo Scientific, Hemel Hemstead, UK). The raw data for each sample analysis consisted LC-MS signals identified by *m*/*z*, retention time and ion signal intensity. The full datasets were imported and converted to .mzXML files by MSconvert (ProteoWizard; Software Foundation, San Diego, CA, USA). Then, XCMS was used to extract the ions of the spectra (determines mass-to charge ratios *m*/*z*), detect chromatographic peaks (retention time), assign them to molecular ions or adducts, align peaks across samples and quantify their relative abundance, which is calculated based on peak area ratios from sample peaks. Finally, peak detection, grouping, and retention time correction were recorded into peak tables [26]. Data from lipids and polar metabolites were unavailable for two participants. In total, the full datasets for 95 participants were included in the analysis.

Peak intensities of each sample were corrected for run-order signal correction by using a locally quadratic (loess) regression model to fit the QC values. Metabolites with a coefficient of variation of their QC values superior to 30% after run-order correction were removed from the peak table [27]. Missing values in the peak tables were replaced by using k-nearest neighbour (KNN) method. Tentative identification of key metabolites was achieved by using an in-house library, as well as in the appropriate metabolite databases: LipidSearch software (Thermo Scientific, San Jose, CA, USA), the Human Metabolome data-base (HMDB) [28] and METLIN [29].

### 2.7. Statistical Analyses

#### 2.7.1. Univariate Analysis

The characteristic of the study population was compared using *t*-test. Normality of the data was tested using the Shapiro–Wilk normality test in. Age, BMI, CTx-1, PTH, vitamin D3, and BMD parameters were expressed as mean and standard deviation (SD). Analysis of covariance was performed to adjust for possible confounders using age and BMI. In addition, our objective was to assess whether lipids and amino acids were associated to BMD. Age-adjusted correlation coefficients were calculated for each lipid and polar metabolites. Age and BMI adjusted mean concentrations of each lipid and polar metabolite were calculated in both groups. To assess whether those associations were independent, we evaluated the pairwise correlations between identified lipids and polar metabolites. *p* values were calculated using Benjamini–Hochberg false discovery rate (FDR), where FDR *p*-value < 0.05 was considered as statistically significant. The fold changes in lipids and polar metabolites between BMD groups were performed by parametric test using Metaboanalyst 4.0 web server [30]. Statistical data analysis was performed using R (R 3.3.3, R Foundation for Statistical Computing, Vienna, Austria).

#### 2.7.2. Multivariate Analysis

Prior to multivariate analysis (MVA), different data sets were created (1) an entire cohort containing all samples (*n* = 95) and (2) a subset (*n* = 30), in which only 15 women with osteoporosis and 15 women with normal BMD were included. Both the entire cohort and subset were used for modelling the correlations between BMD and metabolites in SC menopausal women. For MVA, each variable was mean centered and univariate scaled over all the samples and imported to SIMCA-P+ v14.1 software (Umetrics, Umea, Sweden). SIMCA was used to construct an orthogonal partial least squares (OPLS) regression model for analyzing BMD. The supervised method analyzes the linear relationship between BMD and metabolite profiles. In OPLS, the R2X, R2Y, and Q2 (cum) parameters were used for the model evaluation, representing the explanation, fitness and prediction power respectively. R2X is the percentage of all LC-MS response variables explained by the model. R2Y is the percentage of all sample variables explained by the model. Q2 is the percentage of all sample variables predicted by the model [31]. Only one component was extracted to predict membership probability in each group. Then, from the coefficient score plot, a normal probability plot was created and 95% of features were excluded to fit a new model. The objective was to select the most relevant variables from the interaction between the metabolites and femoral neck BMD relative to bone status (step 1, OPLS methods) and to add these variables into a new model to display the contribution of each metabolite to the modulation of osteoporosis (step 2).

## 3. Results

This study analyzed lipid and polar metabolites in blood plasma of SC menopausal women. Overall, after analyzing lipid and polar metabolites, two data sets were created for further multivariate analysis. The correlations between those compounds and femoral neck BMD were investigated by using univariate and OPLS regression statistical analyses. The lipid profiles revealed differences in plasma of women with normal BMD and lower BMD/ osteoporosis. Polar metabolites exhibited no differences; however, a trend was observed in several metabolites such as a peptide, amino acids, and amines.

### 3.1. Characteristics of the Menopausal Women Bone Status

Women with low BMD were older and had lower BMI when compared with women in the normal group. PTH concentrations were lower in the menopausal groups with low BMD/osteoporosis compared to normal BMD groups. (Table 1). Serum 25(OH) vitamin D_3_ concentrations were adequate (equal to or >50 nmol/L) for all the groups. Bone resorption marker (CTx-1) concentrations were significant higher in the menopausal group with low BMD/ osteoporosis compared to normal BMD groups.

#### 3.1.1. Entire Cohort

Table 1 shows the population characteristics for the menopausal women. The mean ages for these groups were 59.4 and 61.3 for the normal and low BMD groups, respectively. Women with low BMD had lower BMI than those with normal BMD (23.8 vs. 22.5 kg/m^2^, *p* = 0.04) There were no significant differences between the groups for PTH and 25(OH) vitamin D_3_ concentrations. Mean PTH was 4.7 and 4.5 pmol/L (*p* = 0.29) and mean 25(OH) vitamin D_3_ concentrations were 57.4 and 60.1 nmol/L (*p* = 0.23) for the normal and low BMD groups, respectively. However, CTx-1 concentrations and femoral neck BMD were significant different. Mean CTx-1 concentrations were 0.44 and 0.55 ug/L for the normal versus low BMD group. Femoral neck BMD was 0.75 g/cm^2^ and 0.60 g/cm^2^ (*p* ≤ 0.001) for the normal and low BMD groups, respectively.

#### 3.1.2. Subset

Women with osteoporosis were older than those with normal BMD (58 vs. 61, *p* = 0.02), and had lower BMI (23.8 vs. 20.7 kg/m^2^, *p* = 0.008). Similarly, there were no significant differences between the groups for PTH, CTx-1, and 25(OH) vitamin D_3_ concentrations. Mean PTH was 4.8 and 4.3 pmol/L (*p* = 0.08) and mean 25(OH) vitamin D_3_ concentrations were 56.1 and 54.8 nmol/L (*p* = 0.81) for the normal BMD and osteoporosis groups, respectively. Serum CTx-1 concentrations were 0.41 and 0.64 ug/L (*p* = 0.04) and femoral neck BMD was 0.78 g/cm^2^ and 0.51 g/cm^2^ (*p* < 0.001) for the normal BMD and osteoporosis groups, respectively.

### 3.2. Untargeted Analysis Metabolomic Approach

#### 3.2.1. Lipids

##### Entire Cohort

Overall, 7082 features (positive and negative ionization) were detected by lipidomics and after filtration and removal of noise and unstable compounds, the remaining 1662 lipid features were fitted within the OPLS model. The OPLS model was fitted and the cumulative R2Y and Q2 values were 0.302 and 0.042, respectively (Table 2). However, the predictive ability for the model Q2 was low, which indicated that it was not a good model. Therefore, the OPLS model with 1662 features was not able to explain the association of the explanatory variables and femoral neck BMD.

**Figure 1:**
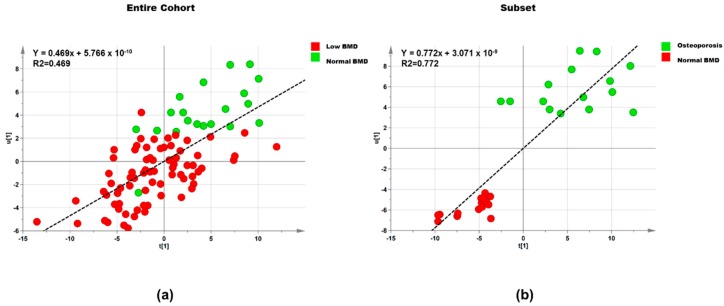
Orthogonal partial least squares (OPLS). OPLS scatter plot of SC menopausal women with normal (green circles) and low BMD and osteoporosis (red circles) values based on orthogonally filtered partial square (OPLS) regression model 2. Graph (**a**): 98 lipids among 95 women, entire cohort. Graph (**b**): 149 lipids among 30 women, subset. t[1]/u[1] correlation plot; t1 refers to scores of the first component in independent variables; u1 refers to scores of the first component in responses.

Subsequently, a new model was created by selecting those features based on the exclusion of un-correlated lipid features on the normal probability coefficient score plot (between >0.05 and <0.95%), from model 1 resulting in 98 lipids being selected. The new model yielded R2Y and Q2 values of 0.469 and 0.233, respectively (Table 2). The new OPLS model parameters for fitness and the predictive capability Q2 were better than in the previous model (step 1) and significant based on the cross-validated analysis of variance (CV-ANOVA) (*p* ≤ 0.001). Figure 1a shows the OPLS predictive model for femoral neck BMD of the lipidomic plasma extracts from normal and low BMD menopausal women. A lineal clustering of the low BMD menopausal women occurred on the left hand of the plot, while normal BMD were clustered on the right top hand side. The results indicated that lipids change linearly with femoral neck BMD (R2Y = 0.469). Further, positive correlations values (upper portion of the plot) indicated increased serum lipid concentrations in normal BMD versus low BMD.

We found negative associations between femoral neck BMD and the lipids of menopausal women. Based on univariate analysis, the top 50 features were significant by *t*-tests. However, only 5 features resulted to be known lipids and these were associated to femoral neck BMD (Table 3). The 5 known lipids include 3 phosphatidylserine (PS), 1 diacylglycerol (DG) and 1 plasmenylphosphatidylethanolamine (plasmenyl-PE). The PS species detected comprised 36:1, 33:6 and 29:6, where a decreasing common trend was observed in low BMD group. A correlation matrix revealed negative correlations among the five lipids and femoral neck BMD, PS 36:1 (r = −0.027), PS 33:6 (−0.070), PS 29:6(−0.086), DG 42:4 (r = −0.075), and plasmenyl-PE 38:4 (r = −0.01).

##### Subset

Similarly, an additional OPLS regression model was fitted to the subset of samples (*n* = 30) with the 1662 lipids to analyze the relationship of femoral neck BMD and lipids.

This OPLS model showed the relationship between the predictive values equals to the observed responses and this was described by high values of R2Y and Q2, 0.601 and 0.209 (Table 2). After excluding 95% of the lipids, a new OPLS model (step 2) was fitted with 149 lipids. The scatter plot for this statistical analysis showed a straight line indicating a relationship between the predictive values equal to the observed responses (Figure 1b). This new model showed higher values of R2Y and Q2 0.773 and 0.54 and was significant based on the CV-ANOVA (*p* ≤ 0.001), and also showed a strong linear correlation of R2Y = 0.772. Lipids that were highly associated to the normal and osteoporosis groups through both approaches are listed in Table 4. Twelve known lipids were significant and included 1 phosphatidic acid (PA), 2 ceramide-1phosphates (CerP), 4 phosphatidylserine (PS) species, 3 diacylglycerol (DG) species, 1 phosphatidylethanolamine (PE), and 1 phosphatidylinositol (PI) (Table 4). A correlation matrix revealed negative correlations among eight lipids and femoral neck BMD, while a positive correlation was observed with PS (20:4), PI, CerP (24:0), and PE (Table 4).

#### 3.2.2. Polar Metabolites

##### Entire Cohort

574 features (positive ionisation) were detected by metabolomics. After filtration and removal of unwanted background and unstable compounds, the remaining 127 polar metabolites were fitted in an OPLS regression model. However, cumulative R2Y and Q2 values were 0.223 and −0.371, respectively (Table 5), and the predictive ability for the model Q2 was very low, which indicated that it was not a suitable model.

**Figure 2:**
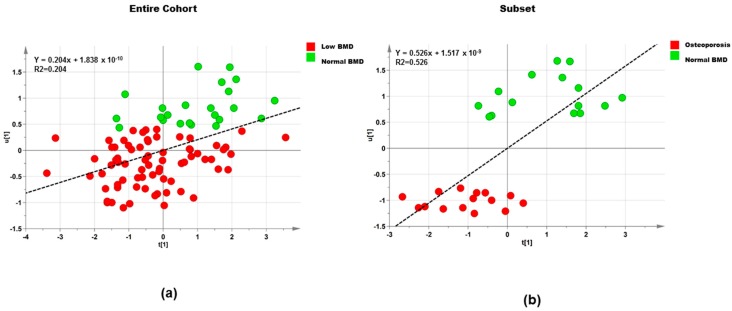
Orthogonal partial least squares (OPLS) scatter plot of polar metabolites, showing relationship between polar metabolites and femoral neck BMD in SC menopausal women with normal (green circles) and low BMD and osteoporosis (red circles) values based on orthogonally filtered partial square (OPLS) regression model 2 with 12 polar metabolites among 95 women, entire cohort (**a**) and 30 women, subset (**b**). t[1]/u[1] correlation plot; t1 refers to scores of the first component in independent variables; u1 refers to scores of the first component in responses.

Features scoring between >0.05 and <0.95% on the normal probability coefficient score plot were taken out to fit a new model (step 2), which had only 12 polar metabolites. Values of cumulative R2Y and Q2 values of for this OPLS model were 0.205 and 0.035, respectively (Figure 2a). Both step 1 and step 2 for polar metabolites showed little prediction for the two OPLS regression models. Therefore, the OPLS model with 127 features was not able to explain the association of the explanatory variables and femoral neck BMD. However, this analysis provided evidence for the association between four polar metabolites and femoral neck BMD (Table 6), 4 aminobutyric acid (r = −0.013), threonine (r = −0.172), a tripeptide formed by asparagine-glycine-cystine (asn-gly-cys) (r = −0.059), and turanose (r = −0.039).

##### Subset

In addition, the OPLS regression (step 1) was fitted to the subset of samples (*n* = 30) with the 127 polar metabolites to analyze the relationship of femoral neck BMD and polar metabolites but this model did not show prediction Q2 = −0.399 (Figure 2b). After filtering using coefficient selection section (as described above) the OPLS model 2 showed a higher cross validation value Q2 = 0.247 when compared with the model from step 1 (Table 5). The correlation matrix revealed negative correlations among lipids and femoral neck BMD, proline (r = −0.295), aminopropionitrile (r = −0.315), threonine (r = −0.170), and asn-gly-cys (r = −0.038); while positive correlation was observed with methionine (r = −0.494) (Table 7).

## 4. Discussion

The potential of metabolomics was explored for the discovery of molecules associated with BMD loss in SC menopausal women. It is well established that bone loss is linked to age and low levels of oestrogen in women in both Caucasian and Asian populations. After menopause, there are several molecular changes affecting bone metabolism, where those alterations result in an increased bone resorption and a declined BMD [32,33]. Thus, the identification of novel potential biomarkers may be useful for understanding the connections of the lipids and polar metabolites associated with osteoporosis and their capacity to predict bone loss in SC menopausal women.

In order to optimize the prediction of biomarkers for bone loss, we created a second data set (subset) to analyze the relationship between the plasma metabolome of SC women with osteoporosis (T-score < −2.5) and normal BMD (T score > −1). Therefore, in this study, we presented results from univariate and multivariate analysis using two different datasets: the entire cohort and a selected subset of samples. The findings were similar in both datasets; however, with the subset analysis, more lipids were identified as being of significance. This statistical filtering using OPLS regression modelling facilitated the removal of un-correlated lipid features and allowed several lipids to be detected as predictors of menopausal osteoporosis in women.

### 4.1. Lipids

In this study, we compared changes of the lipid profiles in SC menopausal women and the relationship of those lipids with femoral neck BMD. Glycerophospholipid species PS (20:4/29:6/31:36/32:6/33:6), PE (42:1), PA (34:4), and PI (14:0) were found to be different between normal BMD and osteoporosis groups.

Lipid metabolism disorders have been linked to pathological conditions including obesity, metabolic syndrome, cardiovascular diseases, and bone loss, where cells and signaling pathways may be affected [34]. Adipocytes and osteoblasts are derived from the mesenchymal stem cells (MSC) and the balance of osteoblast versus adipocyte requires interactions between extracellular signaling stimuli. Changes in any of those factors enhances fat bone deposition and promote bone loss [35]. Oestrogens plays a key role on the cell fate of MSCs to differentiate into either osteoblasts or adipocytes; oestrogen also regulates inflammation [36,37]. Further, peroxisome proliferator-activated receptor (PPARy) transcription factor is essential for adipogenesis. Thus, altered lipid metabolism causes oxidative stress and increased expression of PPARy, which reduces osteoblast number in the skeleton, and oestrogren contributes to the regulation of this PPARy signaling pathway [38,39].

Oestrogen deficiency induces bone loss and changes in lipid profiles, BMD and cytokines; however, little information exists on the application of plasma lipidomics for studying menopausal osteoporosis. In vivo and in vitro studies have reported that lipid metabolism disorders promote bone loss by inhibiting osteoblast differentiation and promoting adipogenic differentiation through MSCs stimuli. Phosphatidylinositol (PI) metabolism is essential for signaling by receptor activator of nuclear factor κB (RANK), a local regulator of osteoclastogenesis and bone resorption [40,41]. Our study showed elevated concentrations of PI species (14:0) in SC women with low BMD/ osteoporosis compared with normal BMD group, suggesting that PI species may lead to chronic inflammatory processes and this might induce bone loss in menopausal osteoporosis. Circulating levels of several lipid classes also have been associated with physiological processes including the regulation of inflammation. In an in vitro study, lipid metabolism of PE, PS, and LysoPC changed during MSCs activation due to pro-inflammatory stimuli with TNF-α and IFN-γ [42]. This suggests that changes in lipid profiles promote production of cytokines and differentiation of osteoclasts and may be attributed to oestrogen withdrawal.

Diacylglycerols (DG) are cellular mediators released from membrane lipids that play a key role in the regulation of inflammation and diseases [43]. Our study showed a decrease in the concentrations of glycerolipid species identified as DG (40:0/42:4) in the osteoporosis group compared to the normal BMD menopausal group. While it is not clear whether DG levels are associated with bone mass there is evidence suggesting a relationship between circulating DG profiles and oestrogen loss. A previous study reported plasma DG (33:2) decreased with ageing [44]. Additionally, an animal study reported serum monoacylglycerol and triacylglycerol concentrations decreased in OVX rats as a model of oestrogen deficiency [45], suggesting that lipid profiles and oestrogen loss upregulate bone-proinflammatory cytokines, which control osteoclast differentiation and promote bone loss.

However, information on the association between plasma lipids and femoral neck BMD in menopausal women is limited. Previous studies in menopausal women have reported conflicting associations between triacylglycerol and hip BMD [34,46,47]. Our findings showed a positive association between DG species and hip femoral neck BMD. This result indicates that lipid profile changes may be involved in MSCs functional, anti-inflammatory activities and cytokine production as a result of oestrogen withdrawal, which enhances MSC signaling and inhibits osteoblast differentiation [48,49,50]. However, further studies are needed to clarify the links between plasma lipid concentrations and femoral neck bone loss in menopausal women.

Sphingolipids also play a structural role in cellular membranes and act as bioactive signalling molecules. Ceramide is one of the simple sphingolipids and is involved in the control of many cellular processes including proliferation, differentiation, and apoptosis [51,52]. It has shown that phosphorylated ceramide (ceramide 1-phosphate) stimulates cell survival and proliferation in bone marrow-derived macrophages through molecules such as NF-κB, RANK, and its ligand RANKL [53,54]. However, in our study two sphingolipid species, CerP (24:0/38:1), were significantly reduced in the osteoporosis group compared with the normal BMD group. Our results contradict a previous animal study of oestrogen loss, where CerP concentrations were upregulated in OVX rats [45]. Furthermore, in a human study, Lee et al. [55] found that higher sphingosine-1-phosphate (S1P) concentrations were associated with low BMD in menopausal women. This suggests that the increased levels of CerP promotes differentiation of bone marrow-derived macrophages with biological effects on bone metabolism and could be attributed to oestrogen deficiency.

Taken together, our findings suggest that altered lipid metabolism could be a regulator of bone cell differentiation and bone loss in SC menopausal women. Further studies are required to investigate the potential role of these lipids as biomarkers for early diagnosis of bone loss in SC menopausal women.

### 4.2. Polar Metabolites

This approach enabled the identification of amino acids, amines, and other polar metabolites. Our study found no significant associations between polar metabolites and femoral neck BMD in SC menopausal groups based on OPLS analysis. However, proline, threonine, and aminopropinitrile concentrations were found to be lower in SC menopausal women with low BMD and osteoporosis based on univariate analysis.

Amino acids play a key role in bone health and are involved in bone remodeling. During osteoporosis, alterations in amino acids may affect bone mass, suggesting that lower levels of circulating amino acids are associated with low BMD. Previous metabolomic studies in menopausal women have reported tryptophan, lysine, homoserine, and 3-hydroxy-l-proline concentrations decreased in the osteoporosis group compared with pre/menopausal women with normal BMD using GC-MS [56]. Miyamoto et al. [20] reported serum concentrations of a dipeptide formed with glycine and glycine (Gly-Gly) and cysteine were lower and hydroxyproline concentrations were higher in low BMD menopausal women using capillary electrophoresis/mass spectrometry (CE-MS). You et al. [22] found higher glutamine concentrations and lower lactate and acetone concentrations associated with low BMD in Taiwanese women using ^1^H NMR Spectroscopy.

Amino acids modulate bone marrow stem cell (BMSC) function, signalling, proliferation, and differentiation in the bone marrow. Arginine is the precursor for the synthesis of many molecules including urea, nitric oxide, proline, and glutamate [57]. Disorders in arginine metabolism are suggested to cause decalcification, disturbance in calcium absorption, and osteomalcia. Growth hormone and insulin-like growth factor-I, both bone-forming growth factors, are stimulated by arginine and a disorder in these factors cause an increase in inflammatory cytokines and osteoporosis [58]. Proline and its metabolite hydroxyproline are the major amino acids components in collagen, and serum hydroxyproline can be used as bone collagen degradation marker [16,59]. Homocysteine is a metabolite in methionine metabolism and at high levels it interferes with collagen cross-linking, suggesting that increased homocysteine levels can lead to increased fracture risk in ageing [60]. Further, aminopropinitrile has been reported in in vitro studies as a collagen cross-linking inhibitor and, with homocysteine, caused decreased bone strength [61]. The mechanistic implications of altered metabolites and their association to bone remodeling and the link to BMD are not clear, but both BMSC differentiation and collagen formation are critical factors that influence BMD, suggesting that perturbations of amino acids under menopausal osteoporosis may partly contribute to bone loss in elderly women. Information on the association between polar metabolites and SC menopausal women is scarce and needs to be corroborated with further work. As we were unable to test for a possible relationship between polar metabolites and femoral neck BMD due to our small study groups and the high variability among participants, our findings need to be kept in perspective.

The limitations of our study include the small number of participants and higher variability among them such as genetic, age, environment, diet and lifestyle, and body composition. However, this was addressed when we analyzed two different datasets, and both the entire cohort and subset findings were consistent as the subset study showed similar compounds to those already detected in the entire cohort. Supporting these findings is the fact the same compounds were associated with BMD in both datasets’ analyses for the pathology of osteoporosis. Overall, future studies on larger populations are needed to confirm these findings and enable more reliable results for assessing the association between metabolome and femoral neck BMD for prognosis of osteoporosis in SC menopausal women.

## 5. Conclusions

This pilot study suggested that plasma lipids and polar metabolites differed between women of normal versus low BMD, and these are involved in several metabolic pathways such as sphingolipid metabolism, phospholipid metabolism, and fatty acid *β*-oxidation. Moreover, this study demonstrates that lipidomic and polar metabolic profiling are promising tools for finding novel biomarkers for bone loss in SC women. Further, prospective studies on larger populations are needed to corroborate these findings and elucidate their key roles in osteoporosis development and progression in SC menopausal women. Thus, understanding the cellular responses to molecular changes in bone metabolism and how these are associated with bone loss in menopausal women may offer the potential of discovering new biomarkers for prognosis of bone loss in elderly women.

## Figures and Tables

**Table 1:** Characteristics of the SC menopausal women according to bone status, entire cohort (*n* = 95) and subset (*n* = 30) analyses.

Parameters	Entire Cohort (*n* = 95)			Subset (*n* = 30)		
	Normal BMD (*n* = 23)	Low BMD (*n* = 72)	*p*-Value	Normal BMD (*n* = 15)	Osteoporosis (*n* = 15)	*p*-Value
Age (years)	59.4 (4.19)	61.3 (4.19)	0.06	58 (3.42)	61 (3.42)	0.02 *
BMI (kg/m^2^)	23.8 (2.61)	22.5 (2.61)	0.04 *	23.8 (2.25)	20.7 (2.25)	<0.001 *
PTH (pmol/L)	4.7 (1.35)	4.5 (1.32)	0.29	4.8 (1.59)	4.3 (1.59)	0.08
CTx-1 (ug/L)	0.44 (0.21)	0.55 (0.20)	0.02 *	0.41 (0.26)	0.64 (0.26)	0.04 *
Vitamin D (nmol/L)	57.4 (15.24)	60.1 (14.87)	0.23	56.5 (16.84)	54.8 (16.84)	0.81
Femoral neck BMD (g/cm^2^)	0.75 (0.05)	0.60 (0.05)	<0.001 *	0.78 (0.04)	0.51 (0.04)	<0.001 *

Data are presented as mean (SD). The resulting *p*-values obtained by ANOVA are given. * *p* < 0.05 was considered as statistically significant.

**Table 2:** Parameters of orthogonal partial least-squares regression models based on the data from lipids separation for the entire cohort and the selected subset of SC menopausal women.

Dataset	Step	Component	R2X	R2Y	Q2
Entire cohort (*n* = 95)	1	1P and O1	0.395	0.302	0.042
Entire cohort (*n* = 95)	2	1P and O2	0.678	0.469	0.233
Subset (*n* = 30)	1	1P and O1	0.434	0.601	0.209
Subset (*n* = 30)	2	1P and O1	0.540	0.773	0.540

P predictive. O orthogonal in X. Component: number of significant component calculated by cross validation. R2X value is the predictive and orthogonal variation in model samples (X) explained by the model. R2Y value is the amount of variation in X which is correlated to Y (response matrix). Q2 value describes the predictive ability of the model.

**Table 3:** Means and 95% confidence interval of lipids associated to the entire cohort of SC menopausal women (*n* = 95) with normal and low femoral neck BMD in univariate and multivariate approaches.

Lipid	Normal BMD ^a^ (*n* = 23)	Low BMD ^a^ (*n* = 72)	*p*-Value	log2 (FC)	Correlation ^b^
PS 31:6; [M + H]^+^	0.893 (0.427–1.871)	0.867 (0.675–1.114)	0.939	−0.455	−0.027
PS 33:6; [M + H]^+^	0.857 (0.408–1.800)	0.876 (0.681–1.127)	0.955	−0.373	−0.070
PS 29:6; [M + H]^+^	0.906 (0.428–1.918)	0.921 (0.714–1.188)	0.967	−0.296	−0.086
DG 42:4; [M + NH4]^+^	0.850 (0.395–1.828)	0.947 (0.725–1.219)	0.804	−0.176	−0.075
Plasmenyl-PE 38:4; [M + H]^+^	0.745 (0.353–1.571)	0.841 (0.653–1.083)	0.760	−0.174	−0.01

Lipids are expressed as mean and 95% confidence interval (CI). ANOVA was used to compare lipid mean concentrations. ^a^ Covariates: age and BMI; ^b^ Age-adjusted correlation coefficient. Statistical *p*-value calculated using Benjamini–Hochberg false discovery rate (FDR). *p*-value < 0.05 was considered as statistically significant. Fold of change (FC) expressed as the relation between the lipid mean of the normal BMD group to the lipid mean of the low BMD group. ^+^ Adducts in positive ionisation. ^–^ Adducts in negative ionisation. PS (phosphatidylserine), DG (diacylglycerol), plasmenyl-PE (plasmenylphosphatidylethanolamines).

**Table 4:** Means and 95% confidence intervals of lipids associated to the subset of selected SC menopausal women (*n* = 30) with normal BMD and osteoporosis in univariate and multivariate approaches.

Lipid	Normal BMD ^a^ (*n* = 15)	Osteoporosis ^a^ (*n* = 15)	*p*-Value	log2 (FC)	Correlation ^b^
PA 34:4; [M − H]^−^	0.531 (0.321–0.878)	1.617 (0.822–3.182)	0.005 *	0.412	−0.403
CerP 38:1; [M + H]^+^	1.871 (1.196–2.927)	0.605 (0.3319–1.105)	0.002 *	−0.637	−0.384
PS 20:4; [M − H]^−^	0.547 (0.330–0.906)	1.585 (0.804–3.124)	0.008 *	0.395	0.274
DG 40:0; [M + NH4]^+^	1.545 (0.907–2.630)	0.425 (0.207–0.870)	0.0029 *	0.729	−0.270
PS 33:6; [M + H]^+^	1.435 (0.908–2.267)	0.504 (0.277–0.933)	0.0046 *	−0.560	−0.363
PS 31:6; [M + H]^+^	1.423 (0.886–2.286)	0.506 (0.267–0.958)	0.0065 *	−0.656	−0.377
PS 32:6; [M + H]^+^	1.414 (0.885–2.259)	0.515 (0.274–0.967)	0.007 *	−0.573	−0.359
PI 14:0; [M − H]^−^	0.608 (0.357–1.036)	1.572 (0.767–3.220)	0.022 *	0.327	0.165
DG 42:4; [M + NH4]^+^	1.510 (0.976–2.336)	0.574 (0.319–1.033)	0.005 *	−0.327	−0.374
CerP 24:0; [M − H]^−^	1.254 (0.728–2.160)	0.418 (0.201–0.869)	0.010 *	−0.265	0.008
DG 47:5; [M + NH4]^+^	1.670 (0.920–3.032)	0.854 (0.382–1.905)	0.135	−0.351	−0.014
PE 42:1; [M − H]^−^	0.592 (0.346–1.013)	1.246 (0.605–2.568)	0.069	0.585	0.034

Lipids are expressed as mean and 95% confidence interval (CI). ANOVA was used to compare lipid mean concentrations. ^a^ Covariates: age and BMI; ^b^ Age-adjusted correlation coefficient; Statistical *p*-value calculated using Benjamini–Hochberg false discovery rate (FDR). * *p*-value < 0.05 was considered as statistically significant. Fold of change (FC) expressed as the relation between the lipid mean of the normal BMD group to the lipid mean of the osteoporosis group. ^+^ Adducts in positive ionisation. ^−^ Adducts in negative ionisation. PS (phosphatidylserine), PA (phosphatidic acid), DG (diacylglycerol), PE (phosphatidylethanolamine), CerP (ceramide-1-phosphate), PE (phosphatidylethanolamine), and PI (phosphatidylinositol).

**Table 5:** Parameters of orthogonal partial least squares regression models based on the data from polar metabolites separation for the entire cohort and the selected subset of SC menopausal women.

Dataset	Step	Component	R2X	R2Y	Q2
Entire cohort (*n* = 95)	1	1P and O1	0.483	0.223	−0.371
Entire cohort (*n* = 95)	2	1P and O1	0.512	0.205	0.035
Subset (*n* = 30)	1	1P and O1	0.437	0.749	−0.399
Subset (*n* = 30)	2	1P and O1	0.514	0.526	0.247

P: predictive. O: orthogonal in X. Component: number of significant component calculated by cross-validation. R2X value is the predictive and orthogonal variation in model samples (X) explained by the model. R2Y value is the amount of variation in X which is correlated to Y (response matrix). Q2 value describes the predictive ability of the model.

**Table 6:** Means and 95% confidence intervals of polar metabolites associated to the entire cohort of SC menopausal women (*n* = 95) with normal and low femoral neck BMD in univariate and multivariate approaches.

Polar Metabolite	Normal BMD ^a^ (*n* = 23)	Low BMD ^a^ (*n* = 72)	*p*-Value	Log2 (FC)	Correlation ^b^
4-Aminobutyric acid	1.400 (0.909–2.155)	0.874 (0.679–1.125)	0.062	−0.185	−0.013
Threonine	1.315 (0.806–2.011)	0.843 (0.658–1.081)	0.073	−0.159	−0.172
Asn–Gly–Cys	0.794 (0.512–1.230)	1.014 (0.512–1.230)	0.337	0.058	0.059
Turanose	1.319 (0.858–2.028)	0.845 (0.657–1.087)	0.076	−0.174	−0.039

Polar metabolites are expressed as mean and 95% confidence interval (CI). ANOVA was used to compare polar metabolites mean concentrations. ^a^ Covariates: age and BMI; ^b^ Age-adjusted correlation coefficient. Statistical *p*-value calculated using Benjamini–Hochberg false discovery rate (FDR). *p*-value < 0.05 was considered as statistically significant. Fold change expressed as the relation between the metabolite mean of the normal BMD group to the metabolite mean of the low BMD group.

**Table 7:** Means and 95% confidence intervals of polar metabolites associated to a subset of selected SC menopausal women (*n* = 30) with normal BMD and osteoporosis in univariate and multivariate approaches.

Polar Metabolite	Normal BMD ^a^ (*n* = 15)	Osteoporosis ^a^ (*n* = 15)	*p*-Value	Log2 (FC)	Correlation ^b^
Proline	1.581 (0.928–2.692)	0.786 (0.384–1.609)	0.084	−0.234	−0.295
Aminopropionitrile	0.729 (0.418–1.272)	1.848 (0.875–3.905)	0.03 *	−0.270	−0.315
Threonine	1.561 (0.911–2.674)	0.765 (0.371–1.579)	0.081	−0.219	−0.170
Methionine	1.534 (0.894–2.632)	0.758 (0.366–1.567)	0.085	0.141	0.494
Asn-Gly-Cys	1.428 (0.782–2.607)	0.593 (0.264–1.334)	0.056	0.142	−0.038

Polar metabolites are expressed as mean and 95% confidence interval (CI). ANOVA was used to compare polar metabolites mean concentrations. ^a^ Covariates: age and BMI; ^b^ Age-adjusted correlation. Statistical *p*-value calculated using Benjamini–Hochberg false discovery rate (FDR). * *p*-value < 0.05 was considered as statistically significant. Fold change expressed as the relation between the metabolite mean of the normal BMD group to the metabolite mean of the osteoporosis group.

## Abbreviations

The following abbreviations are used in this manuscript:

ACN	Acetonitrile
BMD	Bone mineral density
BMI	Body mass index
BMUs	Basic multicellular units
CTx-1	c-terminal telopeptide of type I collagen
DG	Diacylglycerol
DXA	Dual X-ray absorptiometry
EDTA	Ethylenediamine tetraacetic acid
HILIC LC-MS	Hydrophilic interaction chromatography liquid chromatography mass spectrometry
IL-1	Interleukin-1
IK-6	Interleukin-6
CerP	Ceramide-1-phosphate
S1P	Sphingosine-1-phosphate
M-CSF	Macrophage colony-stimulating factor
MSCs	Mesenchymal stem cells
OPLS	Orthogonal partial least squares
OVX	Ovariectomized
PA	Phosphatidic acid
PI	Phosphatidylinositol
Plasmenyl-PE	Plasmenylphosphatidylethanolamine
PE	Phosphatidylethanolamine
PPARy	Peroxisome proliferator-activated receptor
PS	Phosphatidylserine
PTH	parathyroid hormone
ROS	Reactive oxygen species
RP (LC-MS)	Reverse-phase liquid chromatography mass spectrometry
SC	Singaporean-Chinese
TNF-α	Tumor necrosis factor-α
QC	Quality control
WHO	World Health Organization
